# Freeze-thaw treatment effects on the dynamic mechanical properties of articular cartilage

**DOI:** 10.1186/1471-2474-11-231

**Published:** 2010-10-08

**Authors:** Matthew Szarko, Ken Muldrew, John EA Bertram

**Affiliations:** 1 Division of Biomedical Sciences, St. George's, University of London, London, UK; 2 Department of Cell Biology and Anatomy, University of Calgary, Calgary Canada

## Abstract

**Background:**

As a relatively non-regenerative tissue, articular cartilage has been targeted for cryopreservation as a method of mitigating a lack of donor tissue availability for transplant surgeries. In addition, subzero storage of articular cartilage has long been used in biomedical studies using various storage temperatures. The current investigation studies the potential for freeze-thaw to affect the mechanical properties of articular cartilage through direct comparison of various subzero storage temperatures.

**Methods:**

Both subzero storage temperature as well as freezing rate were compared using control samples (4°C) and samples stored at either -20°C or -80°C as well as samples first snap frozen in liquid nitrogen (-196°C) prior to storage at -80°C. All samples were thawed at 37.5°C to testing temperature (22°C). Complex stiffness and hysteresis characterized load resistance and damping properties using a non-destructive, low force magnitude, dynamic indentation protocol spanning a broad loading rate range to identify the dynamic viscoelastic properties of cartilage.

**Results:**

Stiffness levels remained unchanged with exposure to the various subzero temperatures. Hysteresis increased in samples snap frozen at -196°C and stored at -80°C, though remained unchanged with exposure to the other storage temperatures.

**Conclusions:**

Mechanical changes shown are likely due to ice lens creation, where frost heave effects may have caused collagen damage. That storage to -20°C and -80°C did not alter the mechanical properties of articular cartilage shows that when combined with a rapid thawing protocol to 37.5°C, the tissue may successfully be stored at subzero temperatures.

## Background

Degenerative diseases such as osteoarthritis involve deterioration of the extracellular matrix of articular cartilage and results in altered mechanical properties of the tissue. The healing capacity of articular cartilage is limited [1] necessitating the development of methods for repairing or replacing damaged tissue. A current focus within orthopaedics involves the use of either donor tissue or prosthetic devices to replace damaged articular surfaces. Limitations related to implant longevity and donor tissue availability create a need for alternative treatment options [1].

Osteochondral allograft transplantation, involving the implantation of topographically matched, mature osteochondral donor tissue, has been used to treat joint diseases for nearly 100 years [1,2]. Fresh tissue, harvested within 12 hours of donor death and transplanted within three to four days, typically provides the best results for such procedures [3]. However, an increasing population in need of cartilage replacement means that adequate fresh tissue is not always available to meet the demand. Growing concerns regarding infectious disease transmission, problems in matching the size and contour of the defect, and insufficient donor tissue availability all limit the efficacy of fresh allografts [1,3,4]. A potential alternative to the use of fresh replacement tissue is the use of tissue that has been cryogenically preserved. The freezing and cryostorage of osteochondral allografts would ensure tissue sterility and allow for longer-term tissue banking [5]. Effective cryostorage would also enhance tissue availability thereby forming a potentially important method of easing problems in matching donor tissue with patient defects to meet the growing need for transplantable articular cartilage. While many hurdles must be overcome before cyropreservation may be utilized for donor articular cartilage (namely those involved with cell viability), the present investigation attempts to identify the important issue of how freeze-thaw treatment may affect the mechanical properties of articular cartilage.

Although cryostorage of articular cartilage is theoretically an ideal solution, there are practical problems associated with freezing the tissue. Evidence showing the centre of articular cartilage loses heat more slowly than the edges [6] identifies the rate of cooling of articular cartilage stored at subzero temperatures to not be uniform throughout the tissue. Cooling rate disparities may affect ice formation during the freezing of articular cartilage that may result in important mechanical changes. The structure of ice does not allow for the inclusion of impurities such as extracellular solutes. As a result, solutes are advanced away from the growing ice front. If ice progresses faster than the rate of solute movement away from the ice front, a potentially severe osmotic gradient will be created. This osmotic gradient concentrates the solute in the liquid phase just in advance of the ice front that depresses the freezing point immediately in front of the ice interface [7]. Further away from the ice interface the liquid phase has a normal solute concentration with an undepressed freezing point. If the solution temperature near the ice front drops below the undepressed freezing point, constitutional supercooling of the solution may occur and ice crystals are more likely to spontaneously nucleate in this supercooled region [7]. Once formed, an ice crystal attracts surrounding water that, as it freezes, creates a convex shaped ice lens [7,8]. The ice lens may generate mechanical forces that disrupt the non-aqueous cartilage matrix in a manner similar to the mechanical forces present during frost heaves in soils and roadways in colder climates [7,8]. To preserve both the structural and mechanical properties of articular cartilage, an ideal cryopreservation protocol would limit or eliminate the amount of ice crystal formation within the tissue.

Articular cartilage is a viscoelastic tissue with substantial load rate dependence. Although adequate stiffness is necessary for the role of cartilage in weight support, other aspects of cartilage dynamic mechanical properties are equally important to its function, and these are also dependent on load rate. Hysteresis in cyclic loading characterizes the viscous properties of the tissue, and is likely important to the effective functioning of articular cartilage. Characterizing the mechanical properties in allograft articular cartilage is especially important to ensure that the freeze-thaw process, inherent in cryopreservation, does not affect the important time-dependent properties of the tissue. In order to investigate loading rate dependence, in this study a broad range of loading rates (from near zero through 100 Hz) were used to adequately represent the full range of loading rates that the musculoskeletal system is exposed to during locomotion. Investigations into the dynamics of heel strike in locomotion indicate high frequency transients that travel up the skeleton at loading rates up to and including 100 Hz [9-12]. Additionally, recent investigations [13] have found that articular cartilage shows substantial viscoelastic properties when compressed at such loading rates. Dynamic loading over a wide range of frequencies may offer a valuable method for distinguishing mechanical properties of normal and abnormal articular cartilage. The current investigation used low magnitude (28.45 N) dynamic indentation applied over a broad range of frequency as a method for identifying these novel aspects of viscoelastic behaviour of articular cartilage, particularly with respect to understanding the potential effects of cryopreservation and the freeze-thaw process. Cartilage properties were assessed by determining complex stiffness and hysteresis over this range of loading rates after storage for 24 hours at 4, -20°C, -80°C, or snap freezing in liquid nitrogen (196°C) with subsequent storage at -80°C. Although prior studies have tested the effects of a variety of storage temperatures on the mechanical properties of articular cartilage (-20°C, [14,15]; -80°C [1,4,16]; -196°C [17]), to the best of our knowledge, the current investigation is the first to provide a direct comparison of these specific and commonly used storage temperatures. As well, it is the first to investigate how freeze thaw treatment may affect the dynamic mechanical properties on cartilage over such a wide range of loading rates.

## Methods

### Samples

Osteochondral dowels (8 mm diameter with approximately 1 cm attached subchondral bone) were obtained from the medial tibial plateaus of 18-20 month old bovine specimens (n = 17) using a custom hardened-steel coring bit attached to a standard bench-top drill press. Stifle (knee) joints with an undisturbed joint capsule were procured within six hours post mortem. A sample diameter of 8 mm was chosen because it was relatively large compared with the test indenter (1.048 mm diameter), preventing applied loads from being distributed beyond the boundaries of the loading surface. A rounded, semi-spherical indenter tip minimized stress concentration effects at the interface between the indenter and the sample. Overheating and dehydration were prevented during specimen removal by spraying both the coring bit and the cartilage specimen with isotonic phosphate buffered saline (PBS). Each sample was stored in 5 ml PBS at 4°C until testing (48 hours maximum). Such storage did not alter mechanical properties when compared to freshly extracted samples. All experiments were conducted at room temperature (22°C).

### Freeze-Thaw Protocols

Samples were placed in polystyrene culture tubes (VWR) containing 5 ml isotonic PBS solution and stored for 24 hours either at 4°C, -20°C, -80°C, or snap frozen in liquid nitrogen (-196°C) for 2 minutes and subsequently stored at -80°C. The culture tubes (containing samples and PBS) were placed individually by either standing the container in a refrigerator (4°C) freezer (-20°C, -80°C), or individually freezing samples in liquid nitrogen prior to placement at -80°C. Before mechanical testing, each sample (remaining in the culture tube) was rapidly warmed to the testing temperature (22°C) in a 37.5°C saline bath [1,4,16,17]. The total time required for the samples to thermally equilibrate to 22°C were one minute, three minutes, or five minutes, for samples stored at 4°C, -20°C, or -80°C respectively, as determined using a digital thermometer placed in contact with the articular surface of the specimens. While each treatment group consisted of 15 samples, the control group (unfrozen) contained 96 samples, and was used to identify any treatment effects by establishing baseline properties and their variance. Sample positions within the tibial plateau were accounted for by grouping according to limb (right or left) and within plateau (anterior, middle, posterior). Samples from each group (eg. anterior position from the left limb) were then equally divided among the various storage protocols. The different groups were compared using two-way analysis of variance (ANOVA) and no significant differences were found. Mechanical testing occurred twice: immediately after thawing and after four hours of storage at 22°C. The latter mechanical tests were performed to evaluate the permanence of any mechanical property changes resulting from the various temperature treatments.

### Mechanical Analysis

**Figure 1:**
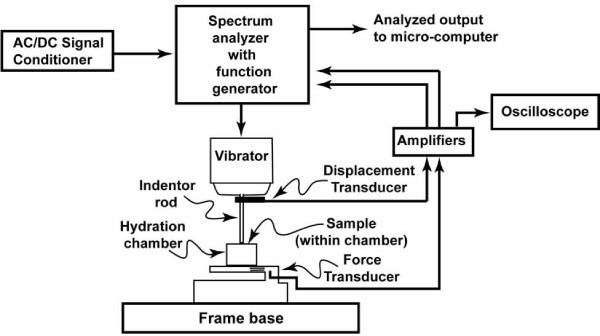
**Diagram illustrating the experimental equipment**. The electrodynamic vibrator (Ling Dynamics, GB) was used to drive the indenter rod. The displacement transducer mounted between the vibrator and rigid indenting rod measured actual displacements of the indenter. The piezoelectric force transducer measured force on the non-articulating side of the sample. The sample was tested while submerged in isotonic PBS. The Stanford SR780 Signal analyzer was used to generate sinusoidal waveforms and analyze voltage signals from the displacement transducer and force beam. The signal amplifier ensured that an adequate signal was produced even though low magnitude forces were used. The oscilloscope was used for identifying pre-load levels and to confirm contact between the indenter and sample surface.

Mechanical properties were assessed by applying a dynamically varying, cyclic compression to the articular surface and comparing this with the output of a load cell supporting the sample. The differences between the phase of the applied waveform and that reaching the support was attributed to the intervening specimen. Movement of the indenter was produced by an electrodynamic vibrator (Ling Dynamics, UK). A displacement transducer mounted between the vibrator and the rigid indenter beam monitored displacement of the indenter and indicated the excitation signal (equal amplitude sinusoids). A custom built piezoelectric force beam, with a resolution of 1 × 10^-4 ^N and a frequency response of 2500 Hz, measured force at the base of the specimen, providing the analysis signal. The electrodynamic vibrator was controlled by the function generator component of a spectrum analyzer (Stanford model SR780). The excitation and analysis signals were then compared using Fast Fourier Transform (FFT) analysis within the spectrum analyzer to determine specimen properties. This arrangement is shown in Figure 1 and has been previously used as reliable, sensitive way to measure dynamic mechanical properties of viscoelastic materials [18]. Maximum indenter displacements reached 0.01 mm creating strains less than 5% and producing loading forces of 28.45 N. Output signals from each transducer were amplified prior to input to the spectrum analyzer to increase resolution.

Two separate dynamic tests were performed to adequately span the loading rate ranges of interest. One was limited to a lower loading rate range (0.1-20 Hz), with input and output signals DC (direct current) coupled to allow low frequency analysis. The other included higher loading rate ranges (30-100 Hz) and was AC (alternating current) coupled to allow filtering of DC drift. Recent studies have shown substantial viscoelastic properties at these higher loading frequencies [13], and reveal an importance of assessing properties at high frequencies. Identifying the mechanical response of articular cartilage to these loading rates, covers the physiologic range of loading that occurs during the heel strike [10-12].

While creating the loading protocol used in the current study, the affect of loading frequency (low frequency testing followed by high frequency testing, vs. high frequency testing followed by low frequency testing) was investigated and found not to influence the results of the mechanical testing. Data was collected after 30 cycles to mechanically condition the specimen and ensure that the viscoelastic steady state properties were assessed (as determined by experimentation during loading protocol creation).

The electrodynamic vibrator motion was driven by sinusoidal waveforms spanning a range of frequencies produced in an exponential chirp pattern. The phases of each sine wave were arranged so that they do not add in phase. The chirp function allowed for the calculation of transfer functions quickly without multiple discrete measurements from single frequency sine waves. This decreased testing time, approximately 3 minutes for each sample and avoided potential changes in tissue properties over the testing period.

Each test began with pre-loading of the cartilage sample with the indenter to 0.1 MPa to ensure that contact between indenter and sample was not lost during dynamic oscillation of the indenter head. The complexities involved with the non-destructive assessment of articular cartilage thickness on intact osteochondral dowels creates an inherent difficulty in calculating applied strain. As a result, the tissue was pre-loaded with a consistent indenter to 0.1 MPa rather than pre-strained to a specific level. After pre-loading, the sample was allowed to reach equilibrium (identified by the signal from the force transducer reaching pre-contact levels) for 5 minutes, previously reported as a reasonable equilibrium time for articular cartilage in standard indentation loading [19]. Each sample was tested in 5 ml of PBS contained by a small plastic cup (with stiffness properties two orders of magnitude greater than cartilage) directly attached to the force beam (this is labeled as the hydration chamber in Figure 1). The ability to maintain continuous contact during the course of each test has been reported by other authors [13] and was confirmed in this study by monitoring real-time oscilloscope traces of displacement transducer and force beam signals. Loss of contact would be quite evident as either complete signal loss from the force beam, or a severely diminished force beam signal indicating that the indenter was moving only the solution around the sample.

Complex stiffness was calculated from the product of the calibration constants of the force and displacement transducers and a transfer function (giving complex moduli) provided by the spectrum analyzer. The complex stiffness function (K*) represents force per unit displacement for the specified frequency [20,21]. The stiffness measurement is complex due to the inclusion of energy loss through hysteresis and consists of both real (storage modulus, *E'*) and imaginary (loss modulus, *E"*) components; E* = *E'*+ *iE*", where E* is the complex modulus and *i *is an imaginary number [20,21]. Hysteresis levels were observed as a shift of the phase relationship between stress and strain waveforms. Hysteresis was calculated by a simple relationship between the storage and loss moduli; tan δ = *E*"/*E*', the tangent of the phase angle. For the purposes of this study, tan δ will be reported in units of degrees.

### Detection of Extracellular Matrix Changes

Blyscan assay (B1000 Biocolor, Ireland), a quantitative dye-binding method that uses 1,9-dimethylmethylene blue to label the sulphated polysaccharide component found in both proteoglycans and the protein-free sulphated GAG chains, was used to measure the total sulphated glycan (with 0.5 μg sensitivity) content within the supernatant surrounding the sample, identifying the potential GAG release due to freeze-thaw treatment. Sircol assay (S1000, Biocolor, Ireland), a quantitative dye-binding method using Sirius Red (Direct Red 80), an anionic dye whose molecules become aligned parallel to the long, rigid structure of native collagens that have intact triple helix organization, was used to quantify the potential for freeze-thaw to break the collagen network and release acid-soluble collagens (with 2.5 μg sensitivity) into the supernatant. These assay techniques have previously been used to identify the extraction of extracellular macromolecules from the tissue [22,23]. The ability of these assays to test the supernatant, avoiding analyses destructive to the sample, allowed samples to be used as their own controls thereby decreasing biological variability and strengthening statistical comparisons.

### Statistical Analyses

Two-way repeated measures analysis of variance (ANOVA) with Tukey post hoc analysis were used to compare treatments and controls, taking into account both storage temperatures and the various loading rate frequencies. One-way ANOVA was used to compare frequency bins within each treatment group. A Bonferroni correction was applied to the loading rates for each treatment over both testing times (9 frequency bin comparisons required a corrected significance level of α = 0.006). A post-hoc power analysis verified the ability of this experimental protocol to distinguish between treatments, finding a statistical power > 0.80 for this study. All statistical methods were conducted using SPSS v.16 (Chicago) and α = 0.05 was accepted as significant for all statistical comparisons unless otherwise noted.

## Results

### Complex Stiffness

**Figure 2:**
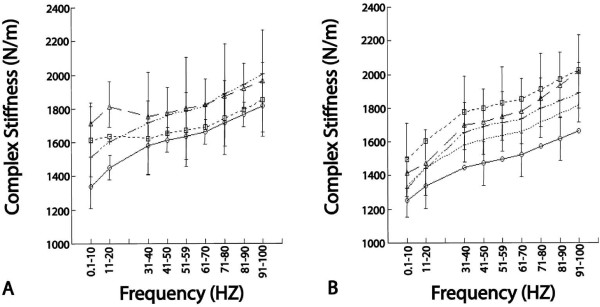
**Complex stiffness (N/m) with standard errors (shown at alternating loading rates for each sample) for the means of the loading rates for control samples at 4°C (identified by open circles) and samples frozen to -20°C (identified by open triangles), -80°C (identified by open squares), or snap frozen in liquid nitrogen and stored at -80°C (identified by crosses)**. Plot **A **identifies the complex stiffness behaviour of samples immediately after thawing and plot **B **identifies the complex stiffness behaviour after 4 hours storage at 22°C. They both reveal no mean complex stiffness differences among the various storage temperatures.

The mean complex stiffness values for treatment and control groups at either testing time were not significantly different (p > 0.3) (Figure 2). Although the stiffness of samples stored at -80°C appears elevated in the second mechanical test, it was not significantly higher (p > 0.5). The consistency in stiffness levels between the 0 hour and 4 hour tests of control samples indicates the non-destructive nature of the testing procedure and the reliability of the mechanical analyses.

### Hysteresis

**Figure 3:**
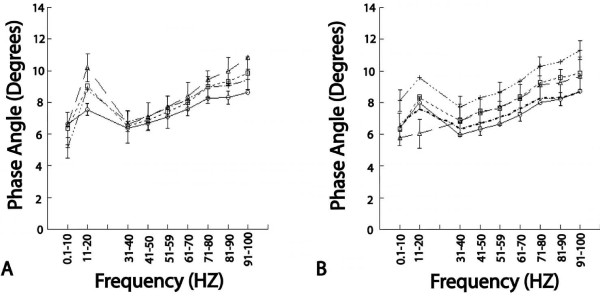
**Hysteresis with standard errors (shown at alternating loading rates for each sample) for the means of the loading rates for control samples at 4°C (identified by open circles) and samples frozen to -20°C (identified by open triangles), -80°C (identified by open squares), or snap frozen in liquid nitrogen and stored at -80°C (identified by crosses)**. Plot **A **identifies the hysteresis of samples immediately after thawing and plot **B **identifies the hysteresis after 4 hours storage at 22°C. This figure shows increased levels of viscous flow over faster loading rate frequencies for samples snap frozen in liquid nitrogen and stored at -80°C (**A **and **B**) and those frozen at -20°C (**B**). The small dotted line in plot B represents the 0 hour control data.

No significant changes (p > 0.5) were found immediately after thawing between samples stored at subzero temperatures and the 4°C controls (Figure 3a). After four hours of storage at room temperature however, those specimens snap frozen in liquid nitrogen and stored at -80°C showed significantly increased (p < 0.01) phase lag values (approximately a 30% increase), most evident over faster loading rates (> 41 Hz) when compared to control specimens. This mechanical property change suggests that this particular freeze-thaw protocol may have caused extracellular matrix damage (Figure 3b). Mean hysteresis values for samples stored at 4°C were similar to each other over both testing times. The consistency in the control samples between testing times again identifies the reliability of the hysteresis measurements and reflects the non-destructive nature of the mechanical testing procedure.

### Detection of Extracellular Matrix Changes

Biochemical assessment of the supernatant solutions for all samples found no differences in glycosaminoglycan or collagen content released from the tissue when compared to unfrozen controls. This suggests that although extracellular matrix changes may have occurred to account for the mechanical differences mentioned above, detectable levels of sulfated glycosaminoglycans or collagen were not released in significantly measurable amounts from the tissue due to freeze-thaw treatment. This is in concordance with previous studies reporting that freeze-thaw treatments do not extract proteoglycans [4].

## Discussion

The current study evaluates the loading rate-dependant mechanical implications of storing articular cartilage at various commonly used storage temperatures, 4°C, -20°C, -80°C, and snap freezing with liquid nitrogen and storage at -80°C. The present results confirm previous findings that storage at -80°C does not change the mechanical properties of articular cartilage [1,4,16], though snap freezing in liquid nitrogen with subsequent storage at -80°C does appear to change the hysteresis properties of articular cartilage.

Stiffness is an important mechanical property of articular cartilage and determines its deflection under load within the joint. Mean complex stiffness appeared unaffected by any of the freeze-thaw treatments used which supports the results of previous investigations studying the potential for subzero storage to alter the mechanical properties of articular cartilage [1,4,14,16]. The absence of detectable stiffness change matches the lack of proteoglycans released into the supernatant (at either time point), as these are a key determinant of the compressive stiffness with the tissue [24].

Hysteresis, a measure of a tissue's time-dependent properties, is particularly important in articular cartilage which is highly viscoelastic. Increased hysteresis, as demonstrated by the phase angle raise in second test of samples snap frozen in liquid nitrogen and stored at -80°C, is indicative of a tissue that will dissipate more energy when loaded. The phase angle increase for these samples was also most evident at higher loading rates (Figure 3b), indicating the importance of evaluating these higher frequencies in order to fully characterize the effects of freeze-thaw treatments. Such changes in material properties likely indicate damage to the collagen network. Previous studies investigating the mechanical effects of collagen disruption have noted increased hysteresis during loading after collagen disruption [9]. The fact that the hysteresis increase in the samples snap frozen in liquid nitrogen and stored at -80°C was evident only after four hours at room temperature may likely be due to the fact that the samples were tested within five minutes of thawing. Storage in PBS for four hours would provide a greater opportunity for the tissue to respond to the presence of collagen damage, likely becoming over-hydrated increasing the tissue's viscous properties. The increased phase angle, combined with the fact that the greatest change occurred over higher loading rates reinforces the conclusion that collagen damage occurred. Collagen has been implicated in the attenuation of loads at higher loading rates [25] while collagen damage leads directly to increases in phase angle [26]. Collagen damage in the samples snap frozen in liquid nitrogen and stored at -80°C is most likely due to ice lens formation. The formation of ice lenses within the extracellular matrix can generate mechanical forces that disrupt the non-aqueous cartilage matrix in a manner similar to the mechanical forces present during frost heaves in soils and roadways in colder climates [7,8]. Rapid freezing through submersion in liquid nitrogen may increase the supercooling of the extracellular fluid, which creates a greater propensity for ice lenses to form [7].

Although assay techniques did not indicate substantial collagen within the supernatant, micro damage to the helical structure of collagen fibres may be enough to alter the mechanical properties of the tissue. The potential for freeze-thaw to disrupt the extracellular matrix and alter the mechanical properties of articular cartilage necessitate further research into the precise interaction between ice crystals and the solid extracellular components of articular cartilage.

## Conclusions

The similar stiffness and hysteresis properties observed between unfrozen controls and samples stored at -20°C as well as those stored at -80°C may indicate that the freezing rate associated with this storage temperature, when matched with rapid thawing at 37.5°C, may limit constitutional supercooling and thus decrease the opportunity for ice lenses to form. The ability to maintain normal mechanical properties in these samples indicates the potential for longer-term cryostorage preservation of this tissue. Cryopreservation of articular cartilage for osteochondral allografting will not be clinically practical until critical issues involving the maintenance of cell viability after cryostorage are solved. However, the present investigation, by identifying freeze-thaw treatments that may damage the tissue as well as those that leave the cartilage mechanics intact, provides a first step in identifying viable storage procedures that may help guide future protocols for subzero storage of articular cartilage in a clinical setting.

## Competing interests

The authors declare that they have no competing interests.

## Authors' contributions

MS participated in the design of the experiment and drafting of the manuscript. MS also carried out the experimentation and analysis of the data. KM participated in the design of the experiment, analysis of data, and drafting of the manuscript. JEAB participated in the design of the experiment, analysis of the data, and drafting of the manuscript. All authors read and approved the final manuscript.

## Pre-publication history

The pre-publication history for this paper can be accessed here:

http://www.biomedcentral.com/1471-2474/11/231/prepub
